# Prediction of response of methotrexate in patients with rheumatoid arthritis using serum lipidomics

**DOI:** 10.1038/s41598-021-86729-7

**Published:** 2021-03-31

**Authors:** Mateusz Maciejewski, Caroline Sands, Nisha Nair, Stephanie Ling, Suzanne Verstappen, Kimme Hyrich, Anne Barton, Daniel Ziemek, Matthew R. Lewis, Darren Plant

**Affiliations:** 1 Computational Systems Immunology, Worldwide Research and Development, Pfizer Inc., New York, USA; 2 National Phenome Centre, Department of Metabolism, Digestion and Reproduction, Imperial College London, London, W12 0NN UK; 3 Versus Arthritis Centre for Genetics and Genomics, Centre for Musculoskeletal Research, Manchester Academic Health Science Centre, The University of Manchester, Oxford Road, Manchester, M13 9PT UK; 4 NIHR Manchester Biomedical Research Centre, Manchester University NHS Foundation Trust, Manchester Academic Health Science Centre, Oxford Road, Manchester, M13 9WL UK; 5 Centre for Epidemiology, Centre for Musculoskeletal Research, Manchester Academic Health Science Centre, The University of Manchester, Oxford Road, Manchester, M13 9PT UK; 6 Division of Musculoskeletal and Dermatological Sciences, The University of Manchester, Manchester, M13 9PT UK

**Keywords:** Bioinformatics, Metabolomics, Machine learning, Lipidomics, Predictive markers, Rheumatoid arthritis, Computational biology and bioinformatics

## Abstract

Methotrexate (MTX) is a common first-line treatment for new-onset rheumatoid arthritis (RA). However, MTX is ineffective for 30–40% of patients and there is no way to know which patients might benefit. Here, we built statistical models based on serum lipid levels measured at two time-points (pre-treatment and following 4 weeks on-drug) to investigate if MTX response (by 6 months) could be predicted. Patients about to commence MTX treatment for the first time were selected from the Rheumatoid Arthritis Medication Study (RAMS). Patients were categorised as good or non-responders following 6 months on-drug using EULAR response criteria. Serum lipids were measured using ultra‐performance liquid chromatography–mass spectrometry and supervised machine learning methods (including regularized regression, support vector machine and random forest) were used to predict EULAR response. Models including lipid levels were compared to models including clinical covariates alone. The best performing classifier including lipid levels (assessed at 4 weeks) was constructed using regularized regression (ROC AUC 0.61 ± 0.02). However, the clinical covariate based model outperformed the classifier including lipid levels when either pre- or on-treatment time-points were investigated (ROC AUC 0.68 ± 0.02). Pre- or early-treatment serum lipid profiles are unlikely to inform classification of MTX response by 6 months with performance adequate for use in RA clinical management.

## Introduction

Without early and effective treatment rheumatoid arthritis (RA) can lead to irreversible joint damage and poor long-term outcomes, including work disability and risk of premature mortality^1^. The treatment pathway for RA starts with synthetic disease-modifying anti-rheumatic drugs (scDMARDs), including methotrexate (MTX)^2^. If disease symptoms are not controlled effectively by MTX, patients are then escalated to more expensive biologic drugs or targeted small molecule inhibitors^3,4^. However, regardless of the class of treatment, each drug comes with a significant non-response rate and it is currently not possible to predict which patient will benefit from treatment with a particular drug^5^.

Although MTX has been licensed for use in RA for more than two decades, few demographic and clinical associations with MTX response have been established. A positive rheumatoid factor titre, high health assessment questionnaire (HAQ) score, a high number of tender joints and higher depression and anxiety before starting on MTX are predictive of non-response after 6 months on drug^6^. However, these factors do not predict MTX non-response perfectly and it is likely that other disease- or patient-related factors also influence treatment outcome. For example, the application of genetic, transcriptomic and proteomic technologies to RA has expanded our understanding of important pathophysiological processes underpinning disease development, progression and response to treatment^5^.

Metabolomics and lipidomics are emergent approaches for studying pathophysiology and patient stratification in RA. Such profiling of biofluids from RA patients has the potential to identify disease processes that underpin important clinical outcomes. For example, serum lipids have been reported to correlate with response to TNFi in RA^7,8^. However, metabolomics studies of MTX response in RA have so far been limited to fewer than 40 patients^9^ and, to our knowledge, no lipidomic specific study applying machine learning algorithms to predict MTX treatment response in RA exists in the literature. The aim of this study was thus to determine if future MTX response can be predicted from ultra-performance liquid chromatography–mass spectrometry (UPLC-MS) derived serum lipidomic data by applying state-of-the-art machine learning methods, under robust nested cross-validation.

## Methods

### Patients and methods

In total, 100 patients were recruited from the Rheumatoid Arthritis Medication Study (RAMS) cohort, a multi-centre 1-year prospective observational study investigating predictors of response to MTX in UK patients with RA. To be eligible for RAMS patients must be over the age of 18 years and have a physician diagnosis of RA or undifferentiated polyarthritis. Since 2009, patients who are about to start MTX for the first time are asked to provide demographic, clinical and psychological data as well as blood samples to permit DNA, RNA and serum-based biomarker studies. Once collected and processed, serum samples were stored in barcode-labelled tubes at − 80 °C and recorded on a fully integrated laboratory management system. Serum was collected from patients at the baseline (pre-treatment) visit and following 4 weeks on treatment. Ethical approval for this study was obtained from the Central Manchester NHS Research Ethics Committee (reference 08/H1008/25), all patients provided written informed consent and all methods were performed in accordance with relevant guidelines and regulations.

### Clinical assessments

Patients were seen by a research nurse prior to commencement of MTX. The baseline clinical assessment included collection of patient demographics and a 28-joint count disease activity score (DAS28)^10^ was measured. Dose of MTX and MTX start date was also recorded. Patients complete a questionnaire including the HAQ. Similar patient-centred data were collected at 6 months following initiation of MTX therapy. The DAS28 was calculated again at 6 months and established EULAR response criteria^11^ were applied to categorize patients into good (n = 50) or non-responders (n = 50).

### Lipid profiling

Serum samples were analysed using ultra-performance liquid chromatography–mass spectrometry (UPLC–MS) following previously described sample preparation, analytical, and quality control (QC) procedures^12,13^. Each set of sample analyses were performed in an order designed to be orthogonal to clinical and demographic data of potential significance with respect to the study design and outcome. Serum samples were prepared as previously described ^12^ for the separation of lipophilic analytes (e.g., complex and neutral lipids) by reversed-phase chromatography (lipid RPC). For quality control and pre-processing, a pooled QC sample was prepared by combining equal parts (25 μL) of each study sample. In brief, 50 μL aliquots were taken from each sample and the pooled QC and diluted 1:1 v/v with ultrapure water. Protein was removed by addition of organic solvent (diluted sample/isopropanol in 1:4 v/v ratio) and a mixture of method-specific authentic chemical standards were added at the protein precipitation step in order to monitor and evaluate data quality during and after the acquisition respectively.

Serum analyses were performed on ACQUITY UPLC instruments (Waters Corp., Milford, MA, USA) coupled to Xevo G2‐S Q-TOF mass spectrometers (Waters Corp., Manchester, UK) via a Z‐spray electrospray ionisation (ESI) source operating in both positive and negative ion modes to generate lipid positive (lipid RPC+) and negative (lipid RPC−) datasets. Lipid separation was conducted using a 2.1 × 100 mm BEH C8 column (Waters Corp., Milford, MA, USA) (further details on the UPLC method and MS parameters can be found in Izzi-Engbeaya et al.^12^). Pooled QC samples were acquired at 10 study sample intervals during sample analyses. An additional set of QC sample dilutions was created from the pooled QC (10 × 100%, 5 × 80%, 3 × 60%, 3 × 40%, 5 × 20%, 10 × 1%) and analysed at the start and end of each set of sample analyses for assessment of analyte response.

Raw data was converted to the mzML open-source format and signals below an absolute intensity threshold of 100 counts were removed using the MSConvert tool in ProteoWizard^14^.

Feature extraction was performed in XCMS^15^ and in-house scripts^16^ applied for elimination of potential run-order effects and feature filtering. Run-order correction was applied at the feature level by fitting a LOESS estimator across intensities of consecutive pooled QC samples and applying to intervening study samples, following an adapted version of the approach proposed by Dunn et al.^17^. Features were filtered to retain only those measured with high analytical quality (relative standard deviation (RSD) in pooled QC < 30%, pooled QC dilution series Pearson correlation to dilution factor > 0.7, RSD in study samples > 1.1* RSD in pooled QC) were retained and put forward for further statistical analysis.

Targeted interrogation of lipids of interest from previous publications^7–9^ was performed in both lipid RPC+ and lipid RPC− datasets. Cholesterol was annotated by matching both retention time and accurate mass to an authentic chemical standard and another four lipids (sn1-LPC 18:3/0:0, sn1-LPC 15:0/0:0, C24H52NO6P and C18H34O2) were tentatively annotated by matching the accurate mass and isotopic pattern/chemical formula of the annotation provided in the publication as no chemical reference standards were available.

### Statistical analysis

#### Assessment of data complexity

Lipid levels were normalised using probabilistic quotient normalization (PQN) before hierarchical cluster analysis. Finite normal mixture modelling and heat maps were used to assess data complexity. The distance matrix required for hierarchical clustering was produced using the complete linkage method (furthest neighbour).The Pearson method was used to compute the correlation matrix required to produce the heat map. Gaussian mixture models, fitted by the expectation–maximization algorithm, were assessed using the Bayesian Information Criterion (BIC).

#### Regression analysis

Linear mixed effect models were developed to detect correlation between individual lipid levels and response to MTX, incorporating the pre-treatment and 4-week sample. Fixed effects included clinical and demographic parameters (i.e. patient sex, age-at-inclusion, MTX start dose, BMI and smoking history) and a patient identifier was included as a random effect. To determine if lipid effects on future treatment response were time-point dependent, an interaction term was specified between time-point and EULAR response. The false discovery rate (FDR) was used to control for Type 1 error at the 5% threshold.

#### Prediction

Machine learning models were built to classify MTX response at 6 months using serum lipid profiles at pre-treatment, following 4 weeks on drug and by taking the difference in serum lipid levels between the 4-week and pre-treatment sample. Models including metabolite levels were compared to models including the following clinical parameters: MTX start dose, steroid use at inclusion, BMI, number of swollen joints, number of tender joints, CRP levels, patients’ assessment of their overall wellbeing, sex, age-at-inclusion, age-at-onset, disease duration, HAQ score and pre-treatment smoking habits. For each contrast, we employed three state-of-the-art machine learning methods: random forest, a kernel-based approach (i.e. radial kernel support vector machine) and regularized logistic regression, a strategy that uses a loss function designed to drop the features that are not predictive. Each model was run under a fivefold nested cross-validation scheme (where hyper-parameters were computed in each of the strata using an inner fivefold cross validation loop, with the default *tuneGrid* parameters in the *caret* library^18^, and a *tuneLength* of 100) to give accurate estimates of predicted performance. Model performance is reported using receiver operating characteristic (ROC) curves; the average ROC area under the curve (AUC) is reported along with the standard errors of the mean (± SE). All ROC AUCs reported throughout were calculated using the out-of-sample cross-validation folds.

## Results

### Samples

Following QC, 3,366 features (1060 in negatively-charged mode and 2306 in positive mode) were available for analysis at pre-treatment and 4 weeks from 100 RA patients categorised as good- (GR, n = 50) or non- (NR, n = 50) responders to MTX following 6 months on drug (Table 1).

**Table 1:** Pre-treatment cohort characteristics.

Baseline characteristics	Eular GR (n = 50)	Eular NR (n = 50)	P-value
Female, n (%)	38 (76)	42 (84)	0.31
Age, me (SD) years	63 (11)	56 (13)	0.004
BMI, me (SD)	27 (5)	29 (6)	0.04
Disease duration, med (IQR)	6.7 (3.7, 13)	9.6 (5.6, 22)	0.07
DAS28, me (SD)	4.6 (0.9)	4.5 (0.8)	0.50
HAQ score, med (IQR)	1.3 (0.6, 1.6)	1.3 (1, 1.9)	0.25
Start dose MTX, med (IQR) mg	15 (10,15)	10 (10,15)	0.02
Current oral steroid use, n (%)	11 (22)	9 (18)	0.60
Smoking n/p/c, %	38/52/10	37/33/30	0.03

Disease duration is presented in months. P-value derived from t-test, Mann–Whitney and chi-squared tests where variables are described by mean, median and n(%), respectively.

*GR* good-responder, *NR* non-responder, *me* mean, *SD* standard deviation, *med* median, *IQR* 25th and 75th percentile, *HAQ* health assessment questionnaire, *MTX* methotrexate, *BMI* body mass index, *DAS28* 28-joint count disease activity score, smoking (n = never, p = past, c = current).

### Data complexity and linear mixed models

No evidence of patient clustering was observed in either the PCA analysis (Supplementary Figure S1), by assessing pair-wise correlation, or using a model-based clustering approach (Supplementary Figures S2, S3). Hierarchical cluster analysis revealed less dissimilarity in within-patient samples (across time-points) compared to between-patient samples (Supplementary Figure S4); median within-patient Pearson correlation = 0.58 (25th and 75th percentile = 0.37 and 0.68, respectively). Linear mixed effects models tested the association between individual lipid levels and EULAR response measured at 6 months. No lipid tested was individually associated with response to treatment (at FDR < 5%). An interaction was specified between sampling time-point and EULAR response to test if the difference in lipid level between responders depended on time-point. No evidence for interaction was observed (at FDR < 5%).

### Classifier performance

**Figure 1:**
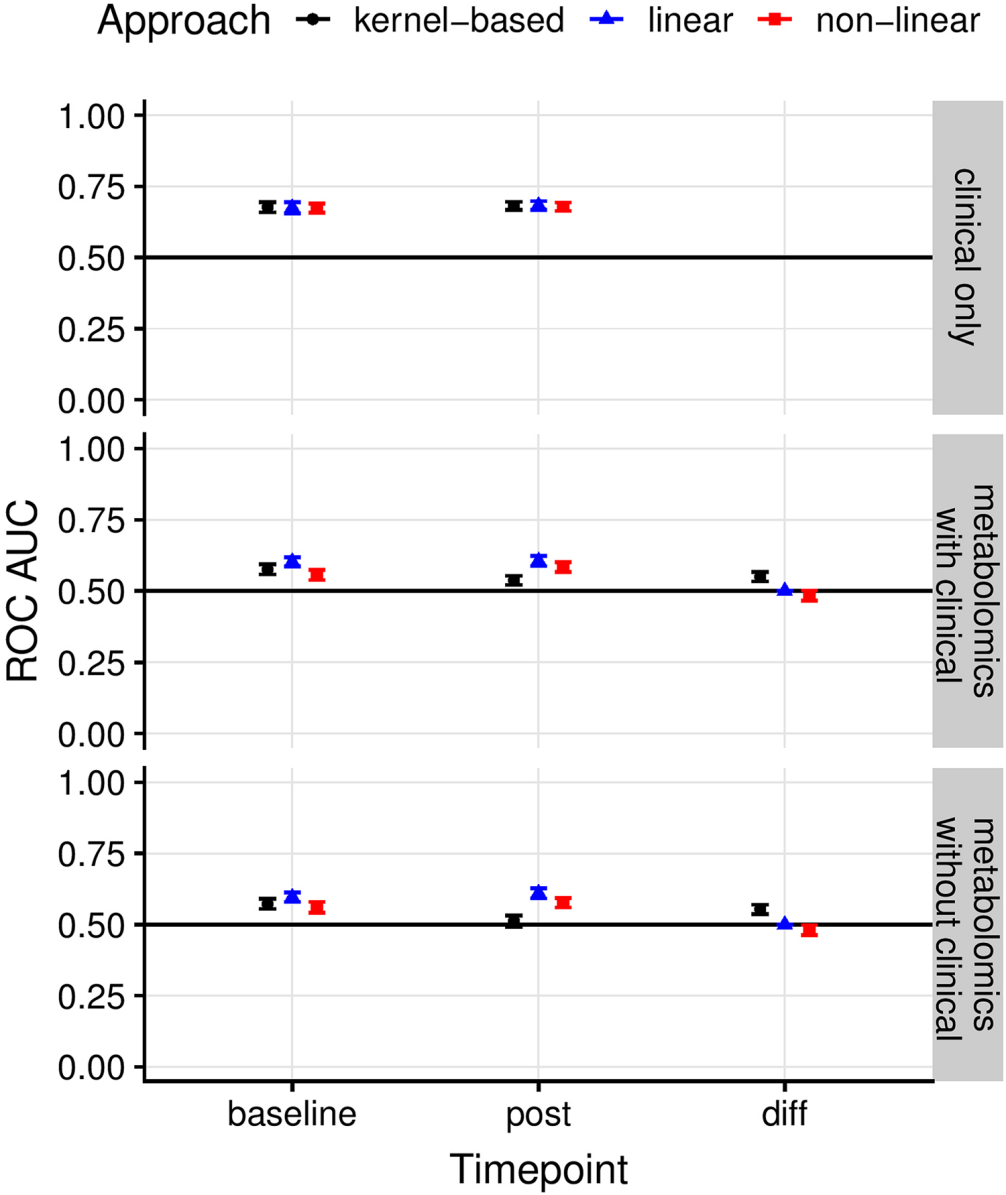
Average ROC AUCs across the cross-validation runs from three machine learning methods including: regularised regression, random forest and a pathway‐supported approach described previously (15). The three methods are labelled linear, non-linear and kernel-based, respectively. Clinical only: performance of models based on clinical data only, recorded at baseline and following 3 months on drug. Metabolomics with clinical: performance of models based on clinical variables plus lipid levels measured at baseline following 4 weeks on drug, and using the ratio of lipid levels between 4 weeks and baseline. Metabolomics without clinical: performance of models based on lipid levels only.

In the models based on lipidomic data very limited predictive utility was observed in either the pre-treatment or 4-week sample or by taking the difference in lipid levels between 4-week and pre-treatment; best performance was observed at the post-treatment time-point for L1/L2-regularised regression models (linear method: ROC AUC 0.61 ± 0.02, Fig. 1). Limited predictive utility was observed in the models based on the clinical data alone, at either baseline or 3 months (Fig. 1). However, the clinical models performed better than the models based on lipid levels.

## Discussion

In this study, one of the largest lipidomic studies of MTX response in RA to date, we applied advanced statistical methods to serum lipidomic profiling data generated at pre-treatment and following 4 weeks on drug in patients receiving MTX for their RA. By assessing the various methods used, we conclude that serum lipid levels present at pre-treatment or during early treatment are unlikely to be useful for classifying future response to MTX in the setting of routine clinical care. None of the metabolites tested were associated with MTX response when tested individually or when considered together by taking a machine learning approach.

To our knowledge, the previous largest metabolomics study of MTX response included 38 patients (25 good-responders and 13 non-responders)^9^. In that small study, on-treatment serum levels of 11 metabolites were found to co-vary with MTX response, measured at the same time-point (24 weeks). Larger and more recent metabolomics study, which included a targeted lipidomic approach, have focussed on TNF-inhibitor (TNFi) treatment response in RA. For example, Cuppen et al.^7^ investigated pre-treatment serum metabolites as potential predictors of TNFi response in 105 RA patients (55 good-responders and 50 non-responders at 6-months). In that study, four metabolites, selected from 139 analysed, improved classification of response (average ROC-AUC 0.84), compared to models including clinical data only (average ROC-AUC 0.72). A study by Tatar et al.^8^ analysed pre-treatment plasma samples from 140 RA patients (100 good-responders and 40 non-responders at 6-months). In our lipid-focussed study we were able to extract peaks for one of the 11 serum metabolites in the study by Wang (cholesterol); two of the four metabolites found to improve classification of response to TNFi in the Cuppen et al. study (sn1-LPC 18:3/0:0 and sn1-LPC 15:0/0:0), and two metabolites detected in the study by Tatar et al. (C24H52NO6P and C18H34O2). None of these metabolites were associated with MTX response in the current study (Supplementary Table S1). Although these findings require replication in independent samples, the fact that these were not found to improve classification of response to MTX in our study implies either a context (i.e. assessment of a later time-point is needed e.g. 24-weeks) or drug specific (i.e. TNFi rather than MTX) utility.

The strengths of the current study include a large sample size (n = 100), robust acquisition of lipidomic data at two time-points (pre-treatment and also during early treatment), rigorous internal model validation by nested cross-validation, a focus on a single drug and profiling of a large number of features (n = 3,366). A limitation to the current study is that modest differences in MTX start dose were observed between responder groups at baseline. However, MTX start dose, along with other important clinical features, was adjusted for in the regression analysis to minimise any potential effects. We do not think modest differences in start dose resulted in altered lipid profiles. If this were the case we would have observed differences when classifying response using models that were built using lipid levels as input. A further limitation is random sampling. Serum samples were taken as part of routine clinical care; therefore, trough sampling was not possible and patients were not asked to fast prior to sample collection. A further limitation was that the on-treatment sampling time-point was arbitrarily decided. As discussed above, serum metabolites have been observed to co-vary with MTX response when later time-points are assessed (i.e. 24-weeks)^9^. It could be that a later time-point may have revealed greater differences than the 4-week sample assessed here. However, the sampling strategy in the current study is in keeping with that performed as part of routine clinical care and the 4-week time-point was chosen because early classification of likely response to MTX was a study aim. Nonetheless, we cannot exclude that better performance may have been achieved if samples were taken at a different time or preprandial following overnight fast. Alternatively, it could be that the lipidome is simply not substantially altered during early MTX therapy for RA. A more broad-scale metabolomics approach might be necessary to improve prediction performance, or a different approach, for example targeting proteomic biomarkers, may be warranted.

## Conclusions

In conclusion, these data do not support the utility of early treatment lipidomic monitoring in routine clinical practice in patients started on MTX for their RA.

## Supplementary Information

Supplementary Information 1.

Supplementary Information 2.

## Data Availability

De-identified lipidomics data presented in this manuscript are available via Mendeley data using the following link: https://data.mendeley.com/datasets/732n8426y5/1.
