# Supplementary material for: Prediction of response of methotrexate in patients with rheumatoid arthritis using serum lipidomics
Source: Sci Rep. 2021 Mar 31;11:7266. doi: 10.1038/s41598-021-86729-7 (PMC8012618; doi:10.1038/s41598-021-86729-7)
Supplement: Supplementary file 1 — Supplementary Information 1. [file 41598_2021_86729_MOESM1_ESM.docx]

**Supplementary material**

Supplementary Table 1: Linear mixed model regression coefficient for previously reported biomarkers of treatment response

| Lipid | Ion mode | Coefficient | Lower 95% CI | Upper 95% CI | p-value | Reference |
| --- | --- | --- | --- | --- | --- | --- |
| sn1-LPC (18:3/0:0) | positive | -0.04 | -0.40 | 0.32 | 0.84 | ^7^ |
| sn1-LPC (15:0/0:0) | positive | 0.15 | -0.17 | 0.47 | 0.37 | ^7^ |
| sn1-LPC (15:0/0:0) | negative | -0.04 | -0.37 | 0.29 | 0.82 | ^7^ |
| C24H52NO6P | positive | -0.24 | -0.60 | 0.11 | 0.19 | ^8^ |
| C18H34O2 | negative | 0.15 | -0.15 | 0.45 | 0.34 | ^8^ |
| Cholesterol | positive | -0.12 | -0.47 | 0.22 | 0.48 | ^9^ |

CI = confidence interval
