# Supplementary material for: Prediction of response of methotrexate in patients with rheumatoid arthritis using serum lipidomics
Source: Sci Rep. 2021 Mar 31;11:7266. doi: 10.1038/s41598-021-86729-7 (PMC8012618; doi:10.1038/s41598-021-86729-7)
Supplement: Supplementary file 2 — Supplementary Information 2. [file 41598_2021_86729_MOESM2_ESM.docx]

**Supplementary Figure 1:** PCA analysis.


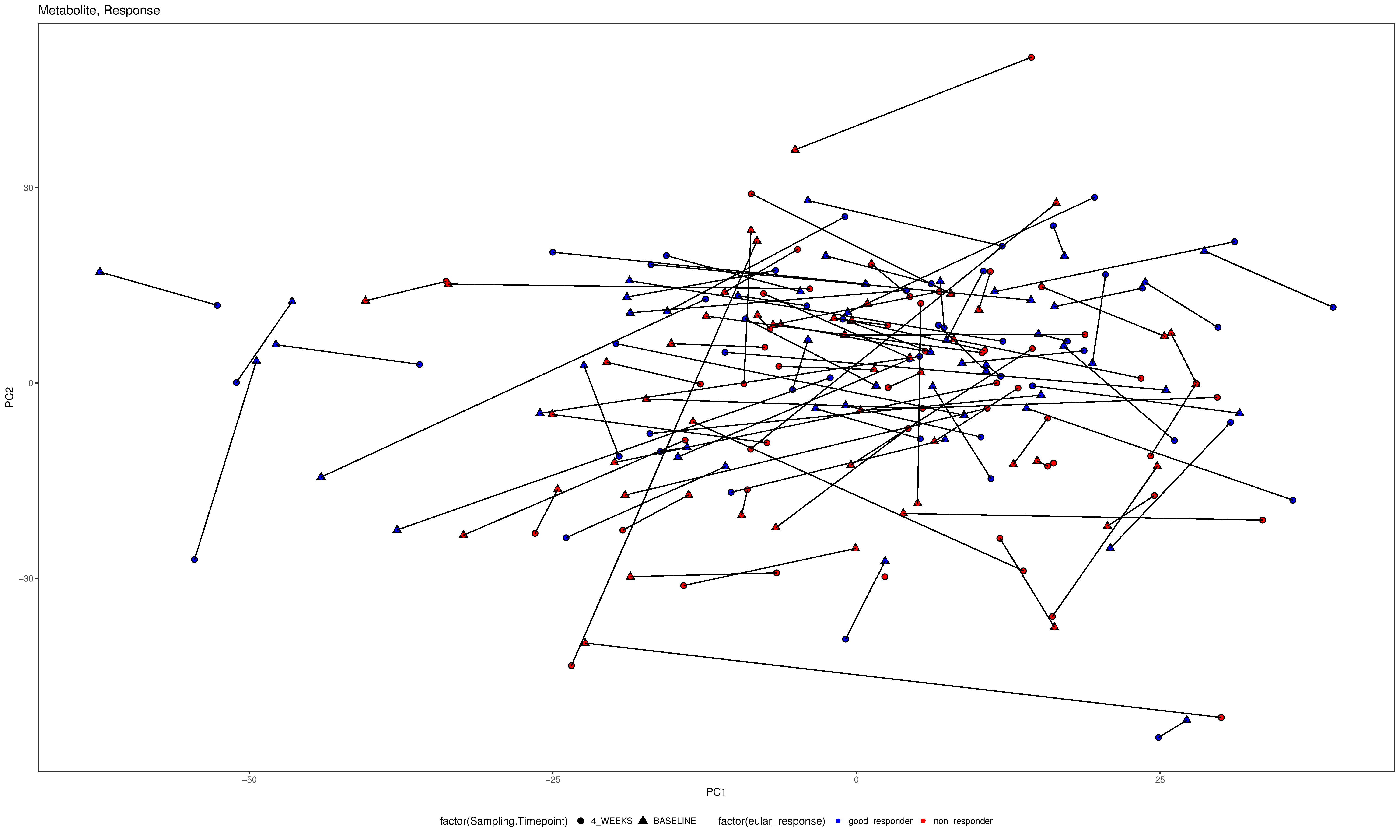


The first and second principal components are plotted. Patient values are colourised according to EULAR response to MTX by 6-months (blue = good responders, red = non-responders). The shape of the data-point indicates the time-point the sample was taken (triangle = baseline sample, circle = 4-week sample).

**Supplementary Figure 2:** Heat map of pair-wise patient correlation calculated using the Pearson’s method.


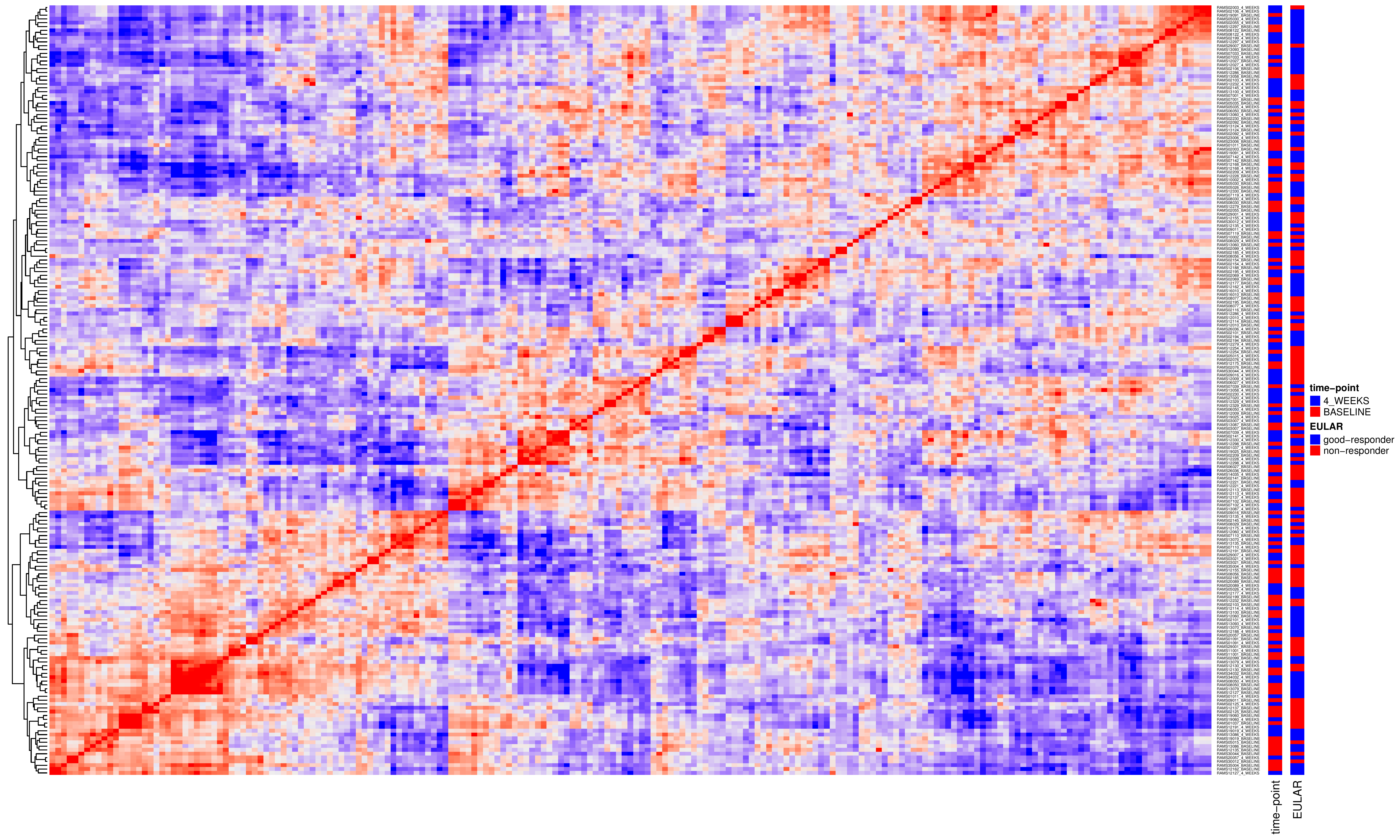


The response status of patients and time-point of sampling is indicated.

**Supplementary Figure 3:** Model-based clustering using Gaussian mixture models, fitted by the expectation–maximization algorithm.


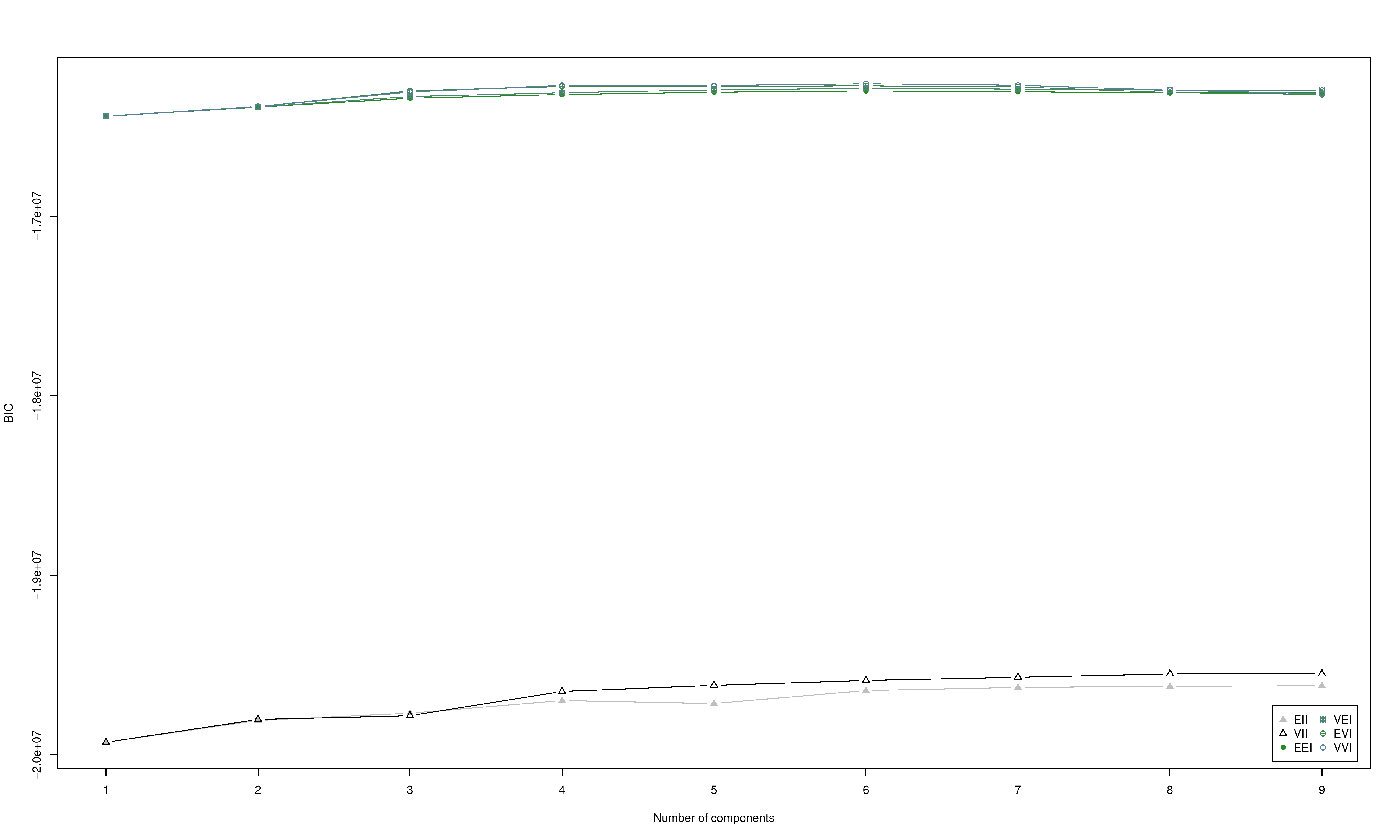


Models were assessed for evidence of patient clustering using the Bayesian information criterion (BIC). Tested models included spherical, equal volume (EII), spherical, unequal volume (VII), diagonal, equal volume and shape (EEI), diagonal, varying volume, equal shape (VEI), diagonal, equal volume, varying shape (EVI) and diagonal, varying volume and shape (VVI). Between 1:9 mixture components (clusters) were specified for which the BIC was calculated.

**Supplementary Figure 4:** Hierarchical cluster of patients and time-points.


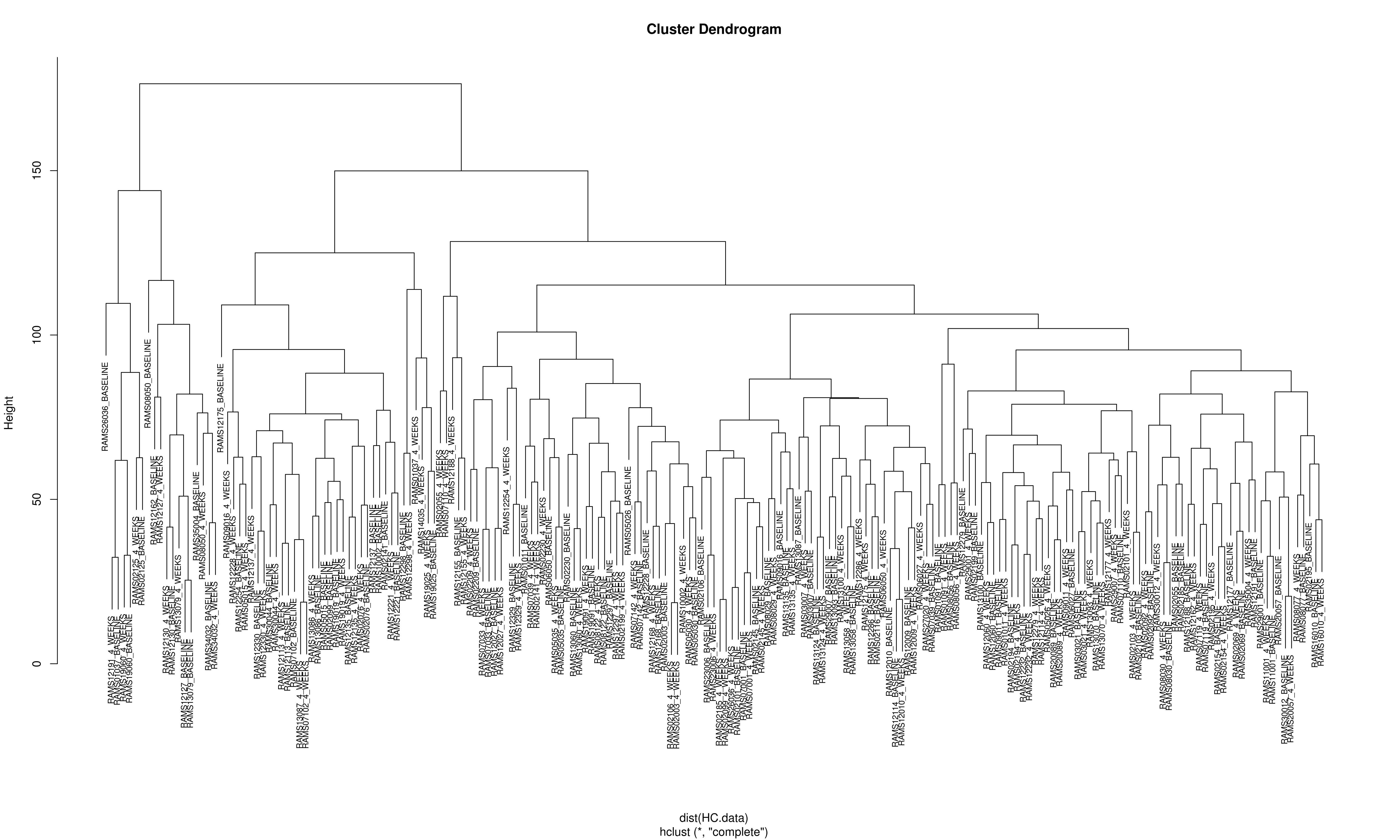


Dissimilarities were computed using the complete linkage method (furthest neighbour).
